# Religiosity and self-rated health among older adults in Colombia

**DOI:** 10.25100/cm.v50i2.4012

**Published:** 2019-06-30

**Authors:** Carlos A Reyes-Ortiz, Claudia Payan, Geraldine Altamar, Fernando Gomez, Harold G. Koenig

**Affiliations:** 1 University of Texas Health Science Center, Houston, Texas, USA.; 2 Florida Agricultural and Mechanical University, Tallahassee, FL, USA; 3 Universidad del Valle, Facultad de Salud, Escuela de Rehabilitación Humana. Cali, Colombia; 4 Universidad del Valle, Facultad de Salud, Escuela de Medicina, Departamento Medicina Familiar, Especialización de Geriatría, Cali, Colombia.; 5 Universidad de Caldas, Facultad de Ciencias para la Salud, Especialización de Geriatría, Manizales, Colombia; 6 Duke University Medical Center, Durham, North Carolina, USA; 7 King Abdulaziz University, Jeddah, Saudi Arabia; 8 Ningxia Medical University, Yinshuan, China

**Keywords:** Religion, rated health, elderly, Colombia, attitude to health, geriatric assessment, spirituality, social class, educational status, aged, ageing, Religión, Autoevaluación de salud, adultos mayores, Colombia, actitud para la salud, valoración geriátrica, espiritualidad, clase social, estatus educativo, envejecimiento, vejez

## Abstract

**Objective:**

To identify the relationship between religiosity and self-rated health among older adults in Colombia.

**Methods:**

Data are drawn from the SABE (Salud, Bienestar y Envejecimiento) Colombia Study, a cross-sectional survey conducted in 2015 involving 18,871 community-dwelling adults aged 60 years and older living in urban and rural areas of Colombia. Religiosity was assessed by self-rated religiosity (how religious are you: not at all, somewhat or very). Self-rated health during previous 30 days was assessed as very good, good, fair, poor or very poor, analyzed as an ordinal variable (1-5) using weighted logistic regression, adjusting for confounders.

**Results:**

Those who were more religious were older, female, had lower socioeconomic status, and were more likely to be married. Multivariate analyses demonstrated that older adults who were more religious had better self-rated health (OR 0.92 95% CI 0.86-0.99, p= 0.038); however, there was a significant interaction effect between gender and religiosity on self-rated health (p= 0.002), such that the relationship between religiosity and health was stronger in men (OR 0.86, 95% CI: 0.79-0.94, p= 0.001) but not significant in women.

**Conclusion:**

Older adults in Colombia who consider themselves more religious, especially men, are less likely to perceive their physical health as poor compared to those who are less religious.

## Resumen

**Objetivo:**

Identificar la relación entre religiosidad y el auto reporte de salud entre adultos mayores en Colombia.

**Métodos:**

Los datos provienen del estudio SABE (Salud, Bienestar y Envejecimiento) Colombia, una encuesta transversal realizada en 2015 en la que participaron 18,871 adultos de 60 años o más que viven en la comunidad y provienen de zonas urbanas y rurales en Colombia. La religiosidad fue autoevaluada (qué tan religiosa eres: nada, algo o muy). La autoevaluación de la salud durante los 30 días previos se evaluó como muy buena, buena, regular, mala o muy mala, analizada como variable ordinal (1-5) usando la regresión logística ponderada, ajustando por factores de confusión.

**Resultados:**

La edad avanzada, el sexo femenino, estar casado, y tener bajo nivel socioeconómico se asociaron con ser más religiosos. Los análisis multivariados demostraron que ser más religioso se asociaba con mejor salud autoevaluada (OR= 0.92; IC 95%= 0.86-0.99; p= 0.038). Sin embargo hubo una interacción significativa entre género y religiosidad sobre la salud autoevaluada (*p*= 0.002), tal que la relación entre religiosidad y salud fue mas fuerte en los hombres (OR= 0.86; IC 95%= 0.79-0.94; *p*= 0.001), pero no fue significativa en mujeres.

**Conclusión:**

Los adultos mayores en Colombia que se consideran a sí mismos más religiosos, especialmente los hombres, tienen menos probabilidades de percibir su salud como pobre en comparación con aquellos que son menos religiosos.

**Remark**

**Table:** 

**1)Why was this study done?**
Self-rated health (SRH) is an important predictor of morbidity and mortality in older adults. We wanted to see whether religiousness was associated with SRH in a large population among Colombian older adults.
**2) What did the researchers do and find?**
A cross-sectional survey conducted in 2015 involving 18,871 community-dwelling adults aged 60 years and older living in the country of Colombia. Older adults in Colombia who consider themselves more religious, especially men, are less likely to perceive their physical health as poor compared to those who are less religious.
**3) What do these findings mean?**
Religiosity is an important resource for older adults. If religiosity improves self-rated health among older adults, or improves the perceptions that they have about their health, then they are more likely to feel better about themselves and may be more likely to seek healthcare services in a more appropriate manner.

## Introduction

Religion is defined as having a religious faith and beliefs, engaging in personal religious practices (e.g., prayer, reading religious scriptures, listening to religious music), and participating in public religious practices (e.g., attending religious services and other religious group activities)^1^ ^-^ ^3^. Religious involvement is associated with better health in older persons ^1^. This is especially true during medical illness when religious involvement has been associated with fewer depressive/anxiety symptoms, more optimism and faster remission from depression in medically ill hospitalized older patients ^4^ ^-^ ^7^. 

Other studies done in Caucasian or African Americans populations have shown that frequent religious activities such as church attendance are associated with an improvement in cancer related or cardiovascular risk factors such as promoting a healthier diet (e.g., more fruits and vegetables consumption) and less risky health behaviors (e.g., smoking, alcohol consumption) ^8^ ^-^ ^11^. Benefits of religiosity have been also seen with respect to protection from colon cancer, reduced functional limitations, and increased disability free life expectancy ^2^ ^,^ ^12^ ^-^ ^15^. 

The association between religious involvement and health is also true in community-dwelling older adults. Women participants in the Nurses’ Health Study (mean age 58) who attended religious services at least once a week had the lowest risk of developing depression, lower suicide rates, and significantly lower all-cause, cardiovascular and cancer mortality ^16^ ^-^ ^18^. In older Mexican Americans, greater religious involvement has been associated with less fear of falling, better cognition, and less influence of depressive symptoms on cognitive functioning ^19^ ^-^ ^21^. 

Self-rated health (SRH) is an important predictor of morbidity and mortality in older adults ^22^ ^,^ ^23^. In Colombia and other Latin American countries, studies have identified several factors associated with SRH in older adults. Gómez *et al*. ^24^, found a correlation between poor SRH with the presence of medical co-morbidity and functional decline. Ocampo *et al*. ^25^, found an association between poor SRH, depression and fear of falling in older adults from Cali. Recently, Parra *et al*. ^26^, found a positive association between perception of neighborhood safety, SRH and quality of life in older adults from Bogota. Likewise, Hambleton *et al*. ^27^, reported an association between negative past socioeconomic events and poor SRH among older Barbadians. Alves *et al*. ^28^, found that higher medical comorbidity and functional decline were associated with poor SRH among older adults in Sao Paulo, Brazil. Finally, Wong *et al*. ^29^, reported an association between poor SRH and functional decline, decreased life satisfaction, poor memory among older adults in Latin America. 

In a SABE study, which included participants from seven Latin American cities, Reyes-Ortiz *et al*. ^30^, found that older adults who indicated that religion was very important were less likely to report fair or poor health compared to those who indicated that religion was only somewhat important or not very important). To our knowledge, no studies have examined the relationship between religion and SRH among older adults in Colombia. The objective of the present study was to identify the relationship between religiosity and self-rated health in a large random sample of older adults from across the country of Colombia.

## Material and Methods

Data were drawn from the 2015 SABE (Salud, Bienestar y Envejecimiento) Colombia Study, a cross-sectional survey of the random sample of older adults, persons age 60 or over, in the country of Colombia. The design of the study and the instrument employed in the SABE Colombia study was derived from the original SABE study conducted in seven Latin American cities ^31^. However, it was adapted to Colombian context. Ethics committees of University of Caldas and University of Valle reviewed and approved the study protocol. Participants provided written informed consent. The survey was administrated in-person in Spanish ^32^.

A multistage random cluster sampling technique was used with stratification of units as follows. First was the selection of municipalities as primary sampling units (PSUs). Second, area segments (i.e., blocks) were identified as secondary sampling units (SSU). Third, a complete listing of all housing units (HUs) was physically located. Finally, household units within a sample HU were selected. The SABE Colombia study was integrated within the general framework of the Colombian National Surveys System, which has a similar sampling design inside the national master sample for country population surveys in Colombia. The estimated sample size was 24,553 individuals, and assuming an 80% response, the original target sample was 30,691. Response rates ranged from 62% in urban areas to 77% in rural sites. The final sample size, including 244 municipalities from all states of the country, was 23,694 ^32^.

Participants were included if they were 60+ years of age, were capable of communicating with the research team, and provided written informed consent. Individuals were excluded if they had a total score of less than 13 on the abbreviated version of the Mini-Mental State Examination (AMMSE) ^33^. For these individuals (17.5% of the sample, n=4,690) a proxy interview was performed, although they were excluded from the present study since questions about religiosity were not asked as part of the proxy interview. Thus, the final sample for this report included 18,871 participants who had complete information on self-rated health and religiosity.

The outcomes were self-rated religiosity and self-rated health. Self-rated religiosity was assessed asking participants how religious they were: none (code=1), somewhat (code=2) or very (code=3), and was analyzed as an ordinal variable (1 to 3, where a higher score indicated greater religiosity). Response options for self-rated health during the previous 30 days were very good (code=1), good (code=2), fair (code=3), poor (code=4) or very poor (code=5); self-rated health was analyzed as an ordinal variable (1 to 5, where a higher scores indicated worse self-rated health). 

Socio-demographic variables included age (years; 60-64, 65-69, 70-74, ≥75), gender (male or female), marital status (married or unmarried), socioeconomic status (SES), and education (years). Other variable was medical comorbidity which was assessed including the presence of seven medical conditions: hypertension, diabetes, coronary heart disease, arthritis, stroke, chronic obstructive pulmonary disease, and cancer. Respondents were asked: “Has a doctor or a nurse told you that you have...?” for each of the conditions listed above. Counts of comorbidities were determined (ranging from 0 to 5) and were analyzed as a continuous variable. 

### Statistical analyses 

To adjust for sampling survey design, data were weighted by using complex survey analyses. Descriptive statistics such as weighted percentages or medians (inter quartile range) were presented, and differences on categorical or ordinal variables were analyzed using the Wald chi-square statistic using the SURVEYFREQ procedure. Graphs were developed describing religiosity across age and gender categories, and self-rated health by gender. Multivariate ordinal logistic regression models examining characteristics associated with religiosity or self-rated health were performed and odds ratios (OR) with confidence intervals (95% CI) were calculated, using the SURVEYLOGISTIC procedure. Relevant interactions terms were tested. Level of statistical significance was set at *p* <0.05. The Statistical Analysis System (SAS), version 9.4 was used for analysis (SAS Institute, Cary, North Carolina, USA).

## Results

Of the 18,871 study participants, 23.0% were age ≥75, 55.8% were female, 43.5% rated their health as fair, 7.5% as poor and 0.8% as very poor; 64.7% considered themselves very religious (Table 1). 

**Table 1:** Characteristics of study population, SABE Colombia Study

Characteristics	Total Population, n=18,871	
	n (%), or median	(inter quartile range)
**Age (years)**	67.3	62.8-73.5
60-64	6,014 (32.1)	
65=69	4,919 (26.2)	
70-74	3,559 (18.7)	
≥75	4,379 (23.0)	
**Female**	10,580 (55.8)	
**Married**	10,676 (56.6)	
**Education (years)**	2.7	0.4-4.8
**SES (levels)**	1.2	1.0-1.9
1 (lowest)	7,759 (41.0)	
2	7,385 (39.2)	
3	3,074 (16.3)	
4	500 (2.7)	
5-6 (highest)	153 (0.8)	
**Comorbidity (number of medical conditions)**	0.6	0.0-1.5
0	5,526 (29.7)	
1	6,464 (34.0)	
2	4,326 (23.0)	
3	1,866 (9.9)	
4	557 (2.7)	
5	132 (0.7)	
**How much religious**		
None	725 (3.9)	
Somewhat	5,857 (31.4)	
Very	12,289 (64.7)	
**Self-rated health**		
Very good	861 (4.5)	
Good	8,195 (43.7)	
Fair	8,209 (43.5)	
Poor	1,439 (7.5)	
Very poor	167 (0.8)	

Weighted data are presented. Comorbidity is the sum score including: hypertension, diabetes, coronary heart disease, arthritis, stroke, chronic obstructive pulmonary disease, and cancer.

SES: Socio Economic state

**Figure 1:**
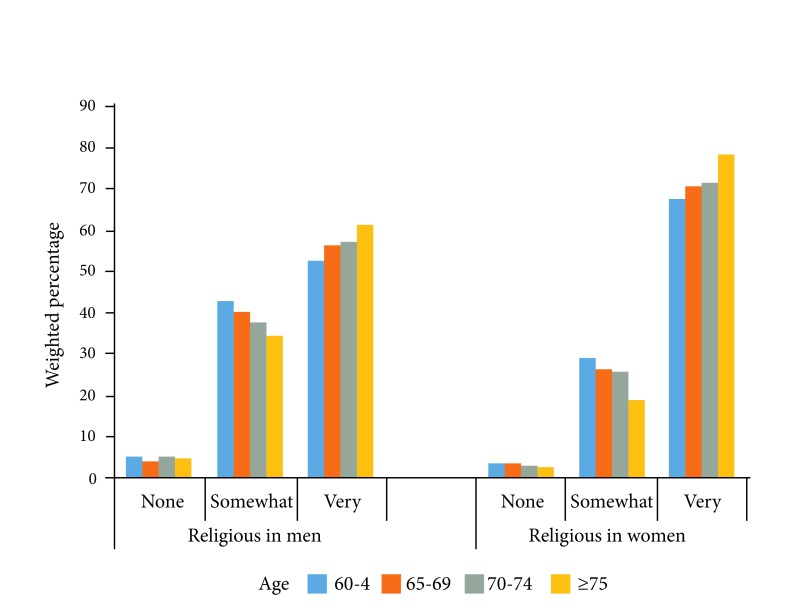
Weighted percentage of religiosity by age and gender

**Figure 2:**
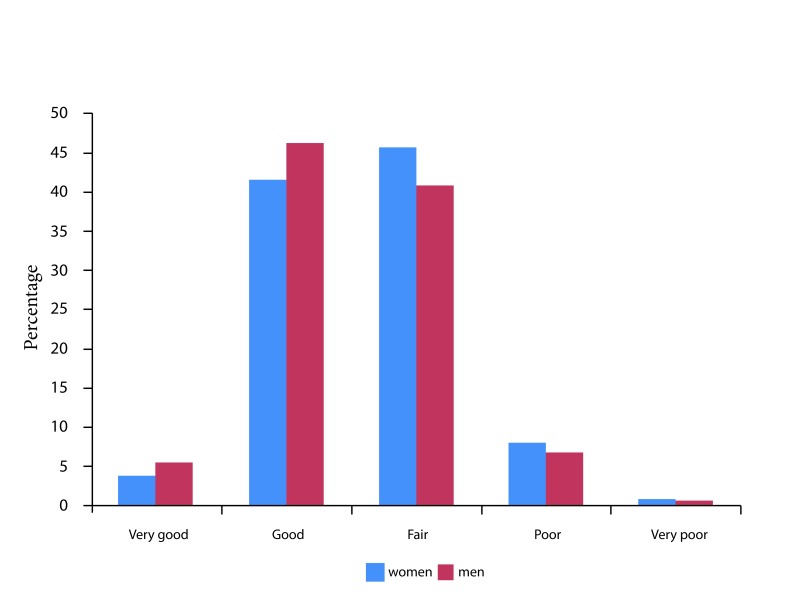
Weighted percentage of self-rated health by gender

Women were more likely to consider themselves very religious compared to men (p <0.0001) (Fig. 1 **)**. Similarly, those who were older were more likely to indicate that they were very religious compared to those who were younger (*p* <0.0001). Women tend to have poorer self-rated health than men (Fig. 2, *p* <0.0001). 

Multivariate analyses demonstrated that religiosity was associated with age, female gender and being married (Table 2). In contrast, higher SES was associated with being less religious. 

**Table 2:** Weighted multivariate logistic regression analyses predicting religiosity,* persons ≥60 years old, SABE Colombia Study, n=18,871**.**

	All n = 18.871		Female n = 10.580		Male n = 8.291	
Characteristic	OR (IC 95%)	valor *p*	OR (IC 95%)	valor *p*	OR (IC 95%)	valor *p*
Age (year)	1.02 (1.02-1.03)	<0.0001	1.03 (1.02-1.04)	<0.0001	1.02 (1.01-1.02)	0.0005
Female (vs male)	1.97 (1.82-2.13)	<0.0001				
Married (vs unmarried)	1.12 (1.03-1.21)	0.0052	1.09 (0.97-1.23)	0.1466	1.18 (1.03-1.35)	0.0151
Education (year)	0.99 (0.98-1.01)	0.5836	1.01 (0.99 1.03)	0.2761	0.98 (0.97 1.00)	0.1313
SES (ordinal) (1 a 4)	0.95 (0.90-0.99)	0.0294	0.92 (0.85-0.98)	0.0169	0.98 (0.92 1.05)	0.5464
Comorbidity (0-5)	1.03 (0.99-1.06)	0.1310	1.02 (0.97-1.07)	0.3521	1.04 (1.09-0.98)	0.1997

* (1-3, greater score is more religious);

OR =odds ratios,

CI =confidence interval.

SES: Socio Economic state

Multivariate analyses indicated that older adults who were more religious had better self-rated health (*p*= 0.038) (Table 3, Model 1). Other factors associated with better self-rated health were higher level of education and SES. By contrast, factors associated with worse health were female gender and higher comorbidity. There was a significant interaction between gender and religiosity on self-rated health (Table 3, Model 2; *p*= 0.002). We did additional adjusted analyses separated by gender where men tend to have better health when being more religious (Table 4, Model 1, OR: 0.86, 95% CI: 0.79-0.94, *p*= 0.001) but the association was not significant in women (Table 4, Model 2). 

**Table 3:** Weighted multivariate logistic regression analysis predicting self-rated health*, persons ≥60 years old, SABE Colombia Study (n=18,871)

	Model 1- Main Effects			Model 2- With Interaction		
Characteristics	OR 95% CI	Estimate (SE)	*p*- value	OR 95% CI	Estimate (SE)	*p*- value
Age (years)	1.00 (0.99-1.00)	0.003 (0.00)	0.2724	1.00 (0.99-1.01)	0.003 (0.00)	0.3069
Female (vs. male)	1.17 (1.07-1.27)	0.078 (0.02)	0.0005		-0.163 (0.07)	0.0322
Married (vs. unmarried)	1.02 (0.94-1.09)	0.008 (0.02)	0.6734	1.02 (0.94-1.09)	0.008 (0.02)	0.6527
Education (years)	0.96 (0.95-0.97)	-0.042 (0.01)	<0.0001	0.96 (0.95-0.97)	-0.042 (0.01)	<.0001
SES (1-5)	0.76 (0.70-0.83)	-0.270 (0.04)	<0.0001	0.76 (0.70-0.83)	-0.270 (0.04)	<.0001
Comorbidity (0-5)	1.53 (1.48-1.58)	0.425 (0.02)	<0.0001	1.53 (1.48-1.58)	0.425 (0.02)	<.0001
Religiosity (1-3)**	0.92 (0.86-0.99)	-0.077 (0.03)	0.0377		-0.056 (0.03)	0.0961
Religiosity*Female					0.093 (0.02)	0.0021

* Ordinal 1 to 5, higher score is worse health; **1=none, 2= somewhat and 3=very religious.

OR= odds ratios;

CI= confidence interval. Estimate is unstandardized beta;

SE=standard error of the estimate.

SES: Socio Economic state

**Table 4:** Weighted multivariate logistic regression analysis predicting self-rated health*, by gender, persons ≥60 years old, SABE Colombia Study

Characteristics	Model 1, Men n=8,291		Model 2, Women n=10,580	
	OR 95% CI	*p*-value	OR 95% CI	*p*-value
Age (years)	1.00 (0.99-1.01)	0.4637	1.00 (0.99-1.01)	0.3350
Married (vs. unmarried)	0.91 (0.82-1.02)	0.1086	1.02 (0.99-1.21)	0.0570
Education (years)	0.94 (0.93-0.96)	<0.0001	0.97 (0.96-0.98)	0.0006
SES (1-5)	0.75 (0.68-0.83)	<0.0001	0.77 (0.70-0.85)	<0.0001
Comorbidity (0-5)	1.51 (1.44-1.58)	<0.0001	1.55 (1.49-1.62)	<0.0001
Religiosity (1-3)**	0.86 (0.79-0.94)	0.0010	1.03 (0.95-1.13)	0.4186

* Ordinal 1 to 5, higher score is worse health; **1=none, 2= somewhat and 3=very religious.

OR= odds ratios;

CI= confidence interval.

SES: Socio Economic state

## Discussion

To our knowledge, this is the first study to examine the relationship between religiosity and self-rated health in older Colombians country-wide. Religiosity was associated with better self-rated health, especially in men. The association was independent of socio-demographic factors, and medical comorbidity. Factors associated with being religious were older age, female gender, being married, and low socioeconomic status. 

Socioeconomic factors and diseases have been considered as the main determinants of self-rated health in the general population as well as in older adults ^22^ ^,^ ^23^ ^,^ ^29^. It has been considered that self-rated health is based on more than just physical health, but also a person’s perception of their psychological, social, and spiritual health ^34^. Several studies have reported that highly religious older adults often have better self-rated health ^35^ ^,^ ^36^. Likewise, studies including minority populations have also reported that religious activities were associated with better perception of health ^37^ ^-^ ^39^. However, several studies based on U.S. Latino populations have not found an association between religious activities and self-rated health ^40^ ^-^ ^42^. The exception, though, is a study among Latin American and Caribbean older adults found a positive association between religiosity and self-rated health ^30^. 

The findings reported here agree with those from other studies that have found religiosity associated with better self-rated health. Koenig *et al*. ^43^, found in medically ill older patients that those categorizing themselves as neither spiritual nor religious tended to have worse self-rated and observer-rated health and greater medical comorbidity. McCullough and Laurenceau ^36^ reported that after controlling for health behaviors, and social support/social activity, women who were highly religious in 1940 had higher mean self-rated health throughout their lifespan, slower rates of linear decline and less pronounced decline in self rated health than did less religious women.

Religiosity has also been associated with positive emotions such as greater life satisfaction, psychological and existential well-being, hope, optimism, and meaning and purpose in life, feelings which help to neutralize the negative emotions that underlie poor self-perceptions of health ^7^ ^,^ ^44^ ^,^ ^45^. The protective effect of religiousness on self-rated health appears to be stronger for people who experience greater suffering. For example, Krause and Bastida ^46^ reported that older Mexican Americans who use their faith to find something positive in the face of suffering tend to rate their health more favorably. In contrast, those who believe that it is important to suffer in silence tend to rate their health less favorably. In some populations, however, religiosity may be related to worse self-rated health ^47^, or is unrelated to it ^48^. 

### Limitations

The findings from this study must be interpreted and generalized with caution. First, the cross-sectional nature of the study does not allow us to determine causality or direction of effect in the relationships observed. Poor self-rated health may just as well lead to less religious involvement, as greater religious involvement may lead to better self-rated health. Second, our measures for religiosity were relatively simple, i.e., subjective indicators of religious importance. 

Nevertheless, the study also has a number of strengths, including the large sample of Colombian older adults who were representative of the urban and rural areas of Colombia; the control for multiple covariates using regression analyses; and as noted earlier, the uniqueness of this study as one of the first to examine religiosity and self-rated health in older Colombians country-wide. 

If religiosity improves self-rated health among older adults, or improves the perceptions that they have about their health, then they are more likely to feel better about themselves and may be more likely to seek healthcare services in a more appropriate manner (rather than seeking medical health for perceived health problems that may be psychological in nature, i.e., psychosomatic, which is true for nearly 50% of medical complaints in primary care settings) ^49^. Religiosity is an important resource for individuals and for population health by facilitating health-related behaviors relevant to older adults especially and beliefs that help them to cope with medical illness or disability ^50^.

## Conclusions

Religious involvement is associated with better self-rated health in older adults, especially men, in the South American country of Colombia. Socioeconomic factors and comorbid illnesses also have a strong influence on self-rated health in older Colombians. Prospective studies are needed to help determine the direction of effect in this relationship, identify mediating factors, and further explore how religiosity may impact self-rated health (and vice-versa). 
