# Supplementary material for: Religiosity and self-rated health among older adults in Colombia
Source: Colomb Med (Cali). 2019 Jun 30;50(2):67–76. doi: 10.25100/cm.v50i2.4012 (PMC6774580; doi:10.25100/cm.v50i2.4012)
Supplement: Translation

# Translation: Religiosidad y el auto-reporte de salud entre adultos mayores en Colombia

**Contribución del estudio**

**Table:** 

**1)Por que se realizó este estudio?**
El auto reporte de salud (ARS) es un importante predictor de morbilidad y mortalidad en adultos mayores. Queríamos ver si la religiosidad estaba asociada con el ARS en una gran población de adultos mayores colombianos.
**2) Cuales fueron los resultados relevantes del estudio?**
Una encuesta transversal realizada en 2015 en la que participaron 18,871 adultos de 60 años de edad o más que viven en la comunidad y que viven en zonas urbanas y rurales de Colombia. Los adultos mayores en Colombia que se consideran más religiosos, especialmente los hombres, tienen menos probabilidades de percibir su salud como pobre en comparación con aquellos que son menos religiosos.
**3) Que Aportan estos resultados?**
La religiosidad es un recurso importante para los adultos mayores. Si la religiosidad mejora el auto reporte de salud entre los adultos mayores, o si mejora las percepciones que tienen sobre su salud, entonces es más probable que se sientan mejor con ellos mismos y es más probable que busquen servicios de atención médica de una manera más apropiada.

## Introducción

La religión se define como tener una fe y creencias religiosas, participar en prácticas religiosas personales (por ejemplo, rezar, leer escrituras religiosas, escuchar música religiosa) y participar en prácticas religiosas públicas (por ejemplo, asistir a servicios religiosos y otras actividades de grupos religiosos) ^1^^-^^3^. La participación religiosa está asociada con una mejor salud en las personas mayores ^1^. Esto es especialmente cierto durante la enfermedad médica cuando la participación religiosa se ha asociado con menos síntomas depresivos / de ansiedad, más optimismo y una remisión más rápida de la depresión en pacientes ancianos hospitalizados con enfermedades médicas ^4^^-^^7^.

Otros estudios realizados en poblaciones caucásicas o afroamericanas han demostrado que las actividades religiosas frecuentes, como la asistencia a la iglesia, están asociadas con una mejora en los factores de riesgo cardiovascular o relacionado con el cáncer, como la promoción de una dieta más saludable (por ejemplo, un mayor consumo de frutas y verduras) y menos comportamientos riesgosos para la salud (por ejemplo, fumar, consumo de alcohol) ^8^^-^^11^. Los beneficios de la religiosidad también se han observado con respecto a la protección contra el cáncer de colon, la reducción de las limitaciones funcionales y el aumento de la esperanza de vida sin discapacidad ^2^^,^^12^^-^^15^.

La asociación entre la participación religiosa y la salud también es válida en los adultos mayores que viven en comunidades. Las mujeres participantes en el Estudio de salud de enfermeras (edad promedio de 58 años) que asistieron a los servicios religiosos al menos una vez a la semana tuvieron el riesgo más bajo de desarrollar depresión, menores tasas de suicidio y una mortalidad general, cardiovascular y por cáncer significativamente menor ^16^^-^^18^. En los americanos de origen mexicano de edad avanzada, una mayor participación religiosa se ha asociado con menos miedo a caerse, mejor cognición y menos influencia de los síntomas depresivos en el funcionamiento cognitivo ^19^^-^^21^.

El auto reporte de salud (ARS) es un importante predictor de morbilidad y mortalidad en adultos mayores ^22^^,^^23^. En Colombia y otros países latinoamericanos, los estudios han identificado varios factores asociados con el ARS en adultos mayores. Gómez *et al*. ^24^, encontraron una correlación entre el ARS deficiente con la presencia de comorbilidad médica y deterioro funcional. Ocampo *et al*. ^25^, encontraron una asociación entre el ARS deficiente, la depresión y el miedo a caerse en adultos mayores de Cali. Recientemente, Parra *et al*. ^26^, encontraron una asociación positiva entre la percepción de seguridad en el vecindario, el ARS y la calidad de vida en adultos mayores de Bogotá. Del mismo modo, Hambleton *et al*. ^27^, informaron una asociación entre eventos socioeconómicos negativos anteriores y el ARS deficiente entre los adultos mayores de Barbados. Alves *et al*. ^28^, encontraron que una mayor comorbilidad médica y deterioro funcional se asociaron con un ARS deficiente entre los adultos mayores en Sao Paulo, Brasil. Por último, Wong et al. ^29^, informaron una asociación entre un ARS pobre y deterioro funcional, disminución de la satisfacción con la vida, y déficit de memoria entre los adultos mayores en América Latina.

En un estudio de SABE, que incluyó participantes de siete ciudades latinoamericanas, Reyes-Ortiz *et al*. ^30^, encontraron que los adultos mayores que indicaron que la religión era muy importante para ellos, tenían menos probabilidades de reportar un ARS deficiente en comparación con aquellos que indicaron que la religión era solo algo importante o no muy importante). Por lo que sabemos, ningún estudio ha examinado la relación entre la religión y el ARS entre los adultos mayores en Colombia. El objetivo del presente estudio fue identificar la relación entre religiosidad y el ARS en una gran muestra aleatoria de adultos mayores de todo el país de Colombia.

## Materiales y Métodos

Los datos se extrajeron del estudio SABE (Salud, Bienestar y Envejecimiento) Colombia 2015, una encuesta transversal de una muestra aleatoria de adultos mayores, personas de 60 años o más, en el país de Colombia. El diseño del estudio y el instrumento empleado en el estudio SABE Colombia se derivaron del estudio original SABE realizado en siete ciudades latinoamericanas ^31^. Sin embargo, se adaptó al contexto colombiano. Los comités de ética de la Universidad de Caldas y la Universidad del Valle revisaron y aprobaron el protocolo de estudio. Los participantes proporcionaron consentimiento informado por escrito. La encuesta fue administrada en persona en español ^32^.

Se usó una técnica de muestreo de grupos al azar de múltiples etapas con la estratificación de unidades de la siguiente manera. Primero fue la selección de los municipios como unidades primarias de muestreo (UPM). En segundo lugar, los segmentos de área (es decir, los bloques) se identificaron como unidades secundarias de muestreo (USM). En tercer lugar, se ubicó físicamente una lista completa de todas las unidades de vivienda (UV). Finalmente, se seleccionaron unidades domésticas dentro de una muestra UV. El estudio SABE Colombia se integró en el marco general del Sistema Nacional de Encuestas de Colombia, que tiene un diseño de muestreo similar dentro de la muestra maestra nacional para las encuestas de población en Colombia. El tamaño de muestra estimado fue de 24,553 individuos, y suponiendo una respuesta del 80%, la muestra objetivo original fue de 30,691. Las tasas de respuesta oscilaron entre 62% en áreas urbanas y 77% en sitios rurales. El tamaño final de la muestra, incluyendo 244 municipios de todos los estados del país, fue de 23,694 ^32^.

Los participantes se incluyeron si tenían más de 60 años de edad, eran capaces de comunicarse con el equipo de investigación y dieron su consentimiento informado por escrito. Los individuos se excluyeron si tenían una puntuación total de menos de 13 en la versión abreviada del Mini-Mental State Examination (AMMSE) 33. Para estos individuos (17.5% de la muestra, n = 4,690) se realizó una entrevista proxy, aunque se excluyeron del presente estudio ya que las preguntas sobre la religiosidad no se hicieron como parte de la entrevista proxy. Por lo tanto, la muestra final para este informe incluyó 18,871 participantes que tenían información completa sobre el ARS y la religiosidad autoevaluada.

Las variables dependientes fueron la religiosidad autoevaluada y el ARS. La religiosidad se evaluó preguntando a los participantes qué tan religiosos eran: nada (código = 1), algo (código = 2) o muy (código = 3), y se analizó como una variable ordinal (1 a 3, donde una puntuación más alta indica mayor religiosidad). Las opciones de respuesta para el ARS durante los 30 días anteriores fue muy bueno (código = 1), bueno (código = 2), regular (código = 3), pobre (código = 4) o muy pobre (código = 5); el ARS se analizó como una variable ordinal (1 a 5, donde las puntuaciones más altas indicaron una salud peor calificada).

Las variables sociodemográficas incluyeron edad (años; 60-64, 65-69, 70-74, ≥75), género (masculino o femenino), estado civil (casado o no casado), estado socioeconómico (SES) y educación (años). Otra variable fue la comorbilidad médica que se evaluó con la presencia de siete afecciones médicas: hipertensión, diabetes, enfermedad coronaria, artritis, accidente cerebrovascular, enfermedad pulmonar obstructiva crónica y cáncer. Se preguntó a los encuestados: “¿Un médico o una enfermera le han dicho que usted tiene ...?” Para cada una de las afecciones enumeradas anteriormente. Se determinaron los recuentos de comorbilidades (rango de 0 a 5) y se analizaron como una variable continua.

### Análisis estadístico

Para ajustar el diseño de la encuesta de muestreo, los datos se ponderaron mediante el uso de análisis complejos para encuestas. Se presentaron estadísticas descriptivas, como porcentajes ponderados o medianas (rango inter cuartil), y se analizaron las diferencias en las variables categóricas u ordinales mediante la estadística de Wald Chi-cuadrado mediante el procedimiento SURVEYFREQ. Se desarrollaron gráficos que describían la religiosidad de acuerdo a categorías de edad y género, y el ARS por género. Se realizaron modelos de regresión logística ordinal multivariada que examinaron las características asociadas con la religiosidad o el ARS y se calcularon los odds ratios (OR) con intervalos de confianza (IC 95%), mediante el procedimiento SURVEYLOGISTIC. Las interacciones relevantes fueron exploradas. El nivel de significación estadística se estableció en *p* <0.05. El Sistema de Análisis Estadístico (SAS), versión 9.4 se utilizó para el análisis (SAS Institute, Cary, Carolina del Norte, EE. UU.).

## Resultados

De los 18,871 participantes del estudio, el 23.0% tenía una edad ≥75, el 55.8% era mujer, el 43.5% calificó su salud regular, el 7.5% como pobre y el 0.8% como muy pobre; el 64,7% se consideraba muy religioso (Tabla 1).

**Tabla 1:** Características de la población del estudio SABE Colombia

Características	Población total, n=18,871	
	n (%), o mediana	(rango inter cuartil)
**Edad (años)**	67.3	62.8-73.5
60-64	6,014 (32.1)	
65-69	4,919 (26.2)	
70-74	3,559 (18.7)	
≥75	4,379 (23.0)	
**Mujer**	10,580 (55.8)	
**Casado**	10,676 (56.6)	
**Educación (años)**	2.7	0.4-4.8
**Niveles socio económicos SES**	1.2	1.0-1.9
1 (mas bajo)	7,759 (41.0)	
2	7,385 (39.2)	
3	3,074 (16.3)	
4	500 (2.7)	
5-6 (mas alto)	153 (0.8)	
**Comorbilidad (numero de condiciones médicas)**	0.6	0.0-1.5
0	5,526 (29.7)	
1	6,464 (34.0)	
2	4,326 (23.0)	
3	1,866 (9.9)	
4	557 (2.7)	
5	132 (0.7)	
**Que tan religioso es**		
Nada	725 (3.9)	
Algo	5,857 (31.4)	
Muy	12,289 (64.7)	
**Auto reporte de salud**		
Muy bien	861 (4.5)	
Bien	8,195 (43.7)	
Regular	8,209 (43.5)	
Pobre	1,439 (7.5)	
Muy pobre	167 (0.8)	

Se presentan datos ponderados. Comorbilidad incluye estas condiciones médicas: hipertensión, diabetes, enfermedad coronaria, artritis, accidente cerebrovascular, enfermedad pulmonar obstructiva crónica y cáncer.

Las mujeres tenían más probabilidades de considerarse muy religiosas en comparación con los hombres (*p* <0.0001) (Fig. 1). Del mismo modo, los que eran muy mayores tenían más probabilidades de indicar que eran muy religiosos en comparación con los que eran más jóvenes (*p* <0.0001). Las mujeres tienden a tener ARS más pobre que los hombres (Fig. 2, *p* <0.0001).

**Figure 1:** Porcentaje ponderado de religiosidad por edad y género

**Figure 2:** Porcentaje ponderado del auto reporte de salud por género

Los análisis multivariados demostraron que una mayor religiosidad estaba asociada con la edad, el género femenino y el ser casado (Tabla 2). En contraste, un mayor SES se asoció con ser menos religioso.

**Tabla 2:** Análisis multivariado ponderado de regresión logística prediciendo religiosidad*, personas ≥60 años, estudio SABE Colombia.

	Todos n = 18.871		Mujeres n = 10.580		Hombres n = 8.291	
Características	OR (IC 95%)	valor *p*	OR (IC 95%)	valor p	OR (IC 95%)	valor *p*
Edad (años)	1.02 (1.02-1.03)	<0.0001	1.03 (1.02-1.04)	<0.0001	1.02 (1.01-1.02)	0.0005
Mujer (vs hombre)	1.97 (1.82-2.13)	<0.0001				
Casado (vs no casado)	1.12 (1.03-1.21)	0.0052	1.09 (0.97-1.23)	0.1466	1.18 (1.03-1.35)	0.0151
Educación (años)	0.99 (0.98-1.01)	0.5836	1.01 (0.99 1.03)	0.2761	0.98 (0.97 1.00)	0.1313
SES (ordinal) (1 a 4)	0.95 (0.90-0.99)	0.0294	0.92 (0.85-0.98)	0.0169	0.98 (0.92 1.05)	0.5464
Comorbilidad (0-5)	1.03 (0.99-1.06)	0.1310	1.02 (0.97-1.07)	0.3521	1.04 (1.09-0.98)	0.1997

* de 1-3, mayor puntuación es más religioso

OR =odds ratios,

, IC = intervalo de confianza.

SES: estado socioeconómico

Los análisis multivariados indicaron que los adultos mayores que eran más religiosos tenían mejor ARS (*p*= 0.038) (Tabla 3, Modelo 1). Otros factores asociados con una mejor salud autoevaluada fueron un mayor nivel de educación y SES. Por el contrario, los factores asociados con una peor salud fueron el sexo femenino y una mayor comorbilidad. Hubo una interacción significativa entre el género y la religiosidad en la salud autoevaluada (Tabla 3, Modelo 2; *p*= 0.002). Hicimos análisis ajustados adicionales separados por género, donde los hombres tienden a tener mejor salud cuando son más religiosos (Tabla 4, Modelo 1, OR: 0.86, IC 95%: 0.79-0.94, *p*= 0.001), pero la asociación no fue significativa en las mujeres (Tabla 4, Modelo 2).

**Tabla 3:** Análisis ponderado de regresión logística multivariada prediciendo el auto reporte de salud *, personas ≥60 años de edad, estudio SABE Colombia (n = 18,871)

	Model 1- efectos principales			Model 2- con interacción		
Caracteristicas	OR 95% CI	Estimate (SE)	*p*- value	OR 95% CI	Estimate (SE)	*p*- value
Edad (años)	1.00 (0.99-1.00)	0.003 (0.00)	0.2724	1.00 (0.99-1.01)	0.003 (0.00)	0.3069
Mujer (vs. hombre)	1.17 (1.07-1.27)	0.078 (0.02)	0.0005		-0.163 (0.07)	0.0322
Casadp (vs. no casado)	1.02 (0.94-1.09)	0.008 (0.02)	0.6734	1.02 (0.94-1.09)	0.008 (0.02)	0.6527
Educación (años)	0.96 (0.95-0.97)	-0.042 (0.01)	<0.0001	0.96 (0.95-0.97)	-0.042 (0.01)	<.0001
SES (1-5)	0.76 (0.70-0.83)	-0.270 (0.04)	<0.0001	0.76 (0.70-0.83)	-0.270 (0.04)	<.0001
Comorbilidad (0-5)	1.53 (1.48-1.58)	0.425 (0.02)	<0.0001	1.53 (1.48-1.58)	0.425 (0.02)	<.0001
Religiosidad (1-3)**	0.92 (0.86-0.99)	-0.077 (0.03)	0.0377		-0.056 (0.03)	0.0961
Religiosidad*mujer					0.093 (0.02)	0.0021

* Ordinal 1 a 5, mayor puntuación es la peor salud; ** 1 = nada, 2 = algo y 3 = muy religioso

OR= odds ratios;

IC = intervalo de confianza. Estimación es no estandarizada

EE = error estándar de la estimación

SES: estado socioeconómico

**Table 4:** Análisis ponderado de regresión logística multivariada prediciendo el auto reporte de salud *, por sexo, personas ≥60 años de edad, estudio SABE Colombia

Características	Model 1, Hombres n=8,291		Model 2, Mujeres n=10,580	
	OR IC 95%	Valor *p*	OR IC 95%	Valor *p*
Edad (años)	1.00 (0.99-1.01)	0.4637	1.00 (0.99-1.01)	0.3350
Casado (vs. no casado)	0.91 (0.82-1.02)	0.1086	1.02 (0.99-1.21)	0.0570
Educación (añoss)	0.94 (0.93-0.96)	<0.0001	0.97 (0.96-0.98)	0.0006
SES (1-5)	0.75 (0.68-0.83)	<0.0001	0.77 (0.70-0.85)	<0.0001
Comorbilidad (0-5)	1.51 (1.44-1.58)	<0.0001	1.55 (1.49-1.62)	<0.0001
Religiosidad (1-3)**	0.86 (0.79-0.94)	0.0010	1.03 (0.95-1.13)	0.4186

* Ordinal de 1 a 5, mayor puntuación es la peor salud; ** 1 = nada, 2 = algo y 3 = muy religioso

OR= odds ratios

IC = intervalo de confianza

SES: estado socioeconómico

## Discusión

A nuestro entender, este es el primer estudio que examina la relación entre religiosidad y ARS el auto reporte de salud en los adultos mayores colombianos en todo el país. La religiosidad se asoció con un mejor ARS, especialmente en los hombres. La asociación fue independiente de factores sociodemográficos, y comorbilidad médica. Los factores asociados con la religión fueron la edad avanzada, el sexo femenino, el ser casado y el bajo nivel socioeconómico.

Los factores socioeconómicos y las enfermedades han sido considerados como los principales determinantes del ARS en la población general, así como en los adultos mayores ^22^^,^^23^^,^^29^. Se ha considerado que el ARS se basa más que en la salud física, sino también en la percepción que tiene una persona de su salud psicológica, social y espiritual ^34^. Varios estudios han informado que los adultos mayores muy religiosos a menudo tienen un mejor ARS 35,36. Asimismo, estudios que incluyen poblaciones minoritarias informaron que las actividades religiosas se asociaron con una mejor percepción de la salud ^37^^-^^39^. Sin embargo, varios estudios basados ​​en poblaciones latinas de EE. UU. no han encontrado una asociación entre actividades religiosas y el ARS ^40^^-^^42^. Sin embargo, la excepción es que un estudio entre adultos mayores de América Latina y el Caribe encontró una asociación positiva entre la religiosidad y el ARS ^30^.

Los hallazgos reportados aquí coinciden con los de otros estudios que han encontrado que la religiosidad se asocia con un mejor ARS. Koenig *et al*. ^43^, encontraron en pacientes ancianos con enfermedades médicas que aquellos que se clasificaban a sí mismos como no espirituales ni religiosos tendían a tener una peor ARS y una mayor comorbilidad médica. McCullough y Laurenceau ^36^ informaron que después de controlar los comportamientos de salud y sociales apoyo / actividad social, las mujeres que eran altamente religiosas en 1940 tenían un mejor promedio de ARS a lo largo de su vida, tasas de declinación lineal más lentas y una disminución menos pronunciada en el ARS que las mujeres menos religiosas.

La religiosidad también se ha asociado con emociones positivas como mayor satisfacción con la vida, bienestar psicológico y existencial, esperanza, optimismo, y sentido y propósito en la vida, sentimientos que ayudan a neutralizar las emociones negativas que subyacen a las malas percepciones de la salud ^7^^,^^44^^,^^45^. El efecto protector de la religiosidad en el ARS parece ser más fuerte para las personas que experimentan un mayor sufrimiento. Por ejemplo, Krause y Bastida ^46^ informaron que los estadounidenses mayores de origen mexicano que usan su fe para encontrar algo positivo frente al sufrimiento tienden a calificar su salud de manera más favorable. En contraste, aquellos que creen que es importante sufrir en silencio tienden a calificar su salud de forma menos favorable. Sin embargo, en algunas poblaciones, la religiosidad puede estar relacionada con una peor autoevaluación de la salud ^47^, o no tiene relación con ella ^48^.

### Limitaciones

Los hallazgos de este estudio deben interpretarse y generalizarse con cautela. Primero, la naturaleza transversal del estudio no nos permite determinar la causalidad o la dirección del efecto en las relaciones observadas. La mala salud autoevaluada también puede conducir a una menor participación religiosa, ya que una mayor participación religiosa puede conducir a una mejor salud autoevaluada. En segundo lugar, nuestras medidas de religiosidad eran relativamente simples, es decir, indicadores subjetivos de importancia religiosa.

Sin embargo, el estudio también tiene varias fortalezas, incluida la gran muestra de adultos mayores colombianos que fueron representativos de las áreas urbanas y rurales de Colombia; el control para variables múltiples mediante análisis de regresión; y como se señaló anteriormente, la singularidad de este estudio como uno de los primeros en examinar la religiosidad y la autoevaluación de la salud en los adultos mayores colombianos en todo el país.

Si la religiosidad mejora la salud autoevaluada entre los adultos mayores, o mejora las percepciones que tienen sobre su salud, entonces es más probable que se sientan mejor con ellos mismos y es más probable que busquen servicios de atención médica de una manera más apropiada en lugar de busca atención médica para problemas de salud percibidos que pueden ser de naturaleza psicológica, es decir, psicosomáticos, lo cual es cierto para casi el 50% de las quejas médicas en entornos de atención primaria ^49^. La religiosidad es un recurso importante para los individuos y para la salud de la población al facilitar la salud las conductas relevantes para adultos mayores especialmente y las creencias que les ayudan a sobrellevar una enfermedad o discapacidad médica ^50^.

## Conclusiones

La participación religiosa puede estar asociada con una mejor salud autoevaluada en adultos mayores, especialmente hombres, en el país sudamericano de Colombia. Los factores socioeconómicos y las enfermedades concomitantes también tienen una fuerte influencia en la salud autoevaluada en los colombianos mayores. Se necesitan estudios prospectivos para ayudar a determinar la dirección del efecto en esta relación, identificar los factores mediadores y explorar más a fondo cómo la religiosidad puede afectar la salud autoevaluada (y viceversa).
